# Complete Genome Sequence of Brucella abortus Phage EF4, Determined Using Long-Read Sequencing

**DOI:** 10.1128/MRA.00212-20

**Published:** 2020-04-30

**Authors:** Calvin Cicha, Jodi Hedges, Ian Novak, Deann Snyder, Mark Jutila, Blake Wiedenheft

**Affiliations:** a Department of Microbiology and Immunology, Montana State University, Bozeman, Montana, USA; Queens College

## Abstract

Brucellaphage EF4 was isolated from elk feces. The 38,321-bp double-stranded DNA genome is predicted to contain 72 coding regions, 38 of which have been assigned predicted functions. This phage displays nucleotide similarity to other brucellaphages of the genus *Perisivirus*.

## ANNOUNCEMENT

*Brucella* species are Gram-negative bacteria and the causative agents of brucellosis, which leads to premature abortions in cattle (1, 2). Brucellosis is a zoonotic disease that can be transmitted from cattle to humans (2). Currently, there is no human vaccine for brucellosis, and *Brucella* species can be cleared only with an extended antibiotic treatment regimen (3). With the need for improved treatment of brucellosis in humans and cattle, phage therapy has been proposed as an alternative treatment (4). Here, we describe the isolation and sequencing of a brucellaphage from the family *Podoviridae* that infects Brucella abortus strain S19.

We isolated and sequenced a bacteriophage infecting Brucella abortus strain S19 from elk fecal samples collected near the Gravelly Mountains in Montana. Elk fecal samples were homogenized in sterile phosphate-buffered saline and filtered through a 0.2-μm filter. Fecal filtrates were combined with the S19 vaccine strain of Brucella abortus and incubated for 30 min at 37°C. Cultures were suspended in 0.75% potato infusion agar (PIA) and poured onto PIA plates. Plates were incubated for 48 h at 37°C. Eleven fecal samples were screened. One plaque (elk fecal 4 [EF4]) was selected for clonal amplification using the plate lysate protocol (5).

**FIG 1:**
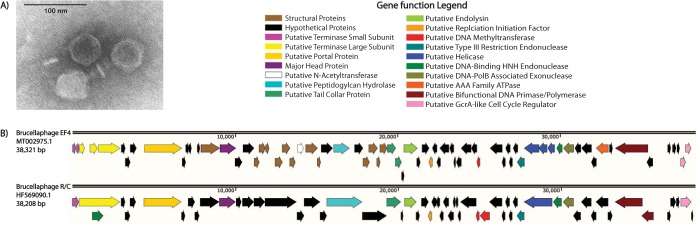
Virion morphology and genome organization of the EF4 phage. (A) Transmission electron microscopy image of brucellaphage EF4, revealing an icosahedral capsid (diameter, 52.5 ± 4.6 nm [*n* = 10]) with a short tail. (B) Genome comparison of brucellaphage EF4 (GenBank accession number MT002975) and brucellaphage R/C (HF569090), revealing similar genome organizations. Gene orientations are indicated with arrows, and functional predictions are colored according to the key.

EF4 phage DNA was isolated and purified via phenol-chloroform extraction (6). DNA was prepared for long-read sequencing using the SQK-LSK109 kit provided by Oxford Nanopore Technologies. DNA was sequenced using the MinION platform with an R9.4.1 FLO-MINSP6 flow cell. Sequencing resulted in a total of 417,284 reads, with an average read length of 1 kb. Quality control and base calling were performed with MinKNOW v19.06.8 and Guppy v3.2.4+d9ed22f, respectively (Oxford Nanopore Technologies). Sequences were assembled using Flye v2.5-g0c3de5b (7). This approach resulted in a single viral contig with 995× coverage. Prokka v1.14.5 was used to identify protein-coding regions within the viral genome (8). Coding regions were queried against the NCBI nonredundant protein database using BLASTp to assign putative functions (Fig. 1) (9). All tools were run with default parameters for long-read analysis.

Brucellaphages have a core genome of 38 kb, with a subset of phages containing two insertions totaling ∼3 kb (10–12). Assembly of the EF4 phage genome produced a single 38,321-bp circular genome (GC content, 48.2%) with neither of the previously described insertions. Since *Perisivirus* genomes are packaged as linear double-stranded DNA but replicate as covalently closed circles, we hypothesize that the circular assembly is a consequence of sequencing both forms of the genome (13). Genome termini were determined via alignment with existing brucellaphages (13). The finding of a single contig with the expected termini and length supports the idea that the whole EF4 phage genome was sequenced. Comparison of the EF4 genome with existing *Perisivirus* genomes using BLASTn revealed a high level of similarity with the genome of brucellaphage R/C (GenBank accession number HF569090), with 99.71% nucleotide identity and similar synteny (10–12).

The EF4 genome has a coding density of 87.8%, with 72 coding sequences (CDSs) identified using the Prokka software package (Fig. 1). Of the 72 predicted CDSs, 38 have sequence similarity to proteins of known function, while the other 34 are similar to hypothetical proteins with no known functions. The 38 CDSs with putative functional assignments include major head, portal, and tail collar proteins, as well as major and minor terminases. This genome also contains expected DNA replication machinery, such as a bifunctional DNA polymerase/primase, helicase, exonuclease, and DNA endonuclease. The genes cluster according to predicted function, with structural and lysis proteins being encoded on the positive strand and DNA replication proteins on the negative strand. This orientation may be involved in regulating gene expression.

### Data availability.

Sequence and related data for the EF4 genome have been deposited under BioProject accession number PRJNA603786, GenBank accession number MT002975, and SRA accession number SRR10985492.
